# Mid-regional pro-adrenomedullin as a prognostic marker in sepsis: an observational study

**DOI:** 10.1186/cc3885

**Published:** 2005-11-15

**Authors:** Mirjam Christ-Crain, Nils G Morgenthaler, Joachim Struck, Stephan Harbarth, Andreas Bergmann, Beat Müller

**Affiliations:** 1 Department of Internal Medicine, University Hospital Basel, Petersgraben 4, 4031 Basel, Switzerland; 2 Research Department, Brahms AG, Hennigsdorf/Berlin, Neuendorfstrasse 25, 16761 Hennigsdorf, Germany; 3 Research Department, Brahms AG, Hennigsdorf/Berlin, Neuendorfstrasse 25, 16761 Hennigsdorf, Germany; 4 Division of Hospital Epidemiology, University Hospital Geneva, 24, rue Micheli-du-Crest, 1211 Geneva 14, Switzerland; 5 Research Department, Brahms AG, Hennigsdorf/Berlin, Neuendorfstrasse 25, 16761 Hennigsdorf, Germany; 6 Department of Internal Medicine, University Hospital Basel, Petersgraben 4, 4031 Basel, Switzerland

## Abstract

**Introduction:**

Measurement of biomarkers is a potential approach to early assessment and prediction of mortality in patients with sepsis. The aim of the present study was to evaluate the prognostic value of mid-regional pro-adrenomedullin (MR-proADM) levels in a cohort of medical intensive care patients and to compare it with other biomarkers and physiological scores.

**Method:**

We evaluated blood samples from 101 consecutive critically ill patients admitted to the intensive care unit and from 160 age-matched healthy control individuals. The patients had initially been enrolled in a prospective observational study investigating the prognostic value of endocrine dysfunction in critically ill patients ("PEDCRIP" Study). The prognostic value of MR-proADM levels was compared with those of two physiological scores and of various biomarkers (for example C-reactive Protein, IL-6, procalcitonin). MR-proADM was measured in EDTA plasma from all patients using a new sandwich immunoassay.

**Results:**

On admission, 53 patients had sepsis, severe sepsis, or septic shock, and 48 had systemic inflammatory response syndrome. Median MR-proADM levels on admission (nmol/l [range]) were 1.1 (0.3–3.7) in patients with systemic inflammatory response syndrome, 1.8 (0.4–5.8) in those with sepsis, 2.3 (1.0–17.6) in those with severe sepsis and 4.5 (0.9–21) in patients with septic shock. In healthy control individuals the median MR-proADM was 0.4 (0.21–0.97). On admission, circulating MR-proADM levels in patients with sepsis, severe sepsis, or septic shock were significantly higher in nonsurvivors (8.5 [0.8–21.0]; *P *< 0.001) than in survivors (1.7 [0.4–17.6]). In a receiver operating curve analysis of survival of patients with sepsis, the area under the curve (AUC) for MR-proADM was 0.81, which was similar to the AUCs for IL-6, Acute Physiology and Chronic Health Evaluation II score and Simplified Acute Physiology Score II. The prognostic value of MR-proADM was independent of the sepsis classification system used.

**Conclusion:**

MR-proADM may be helpful in individual risk assessment in septic patients.

## Introduction

Sepsis is the leading cause of death in critically ill patients in the USA. It develops in 750,000 people annually, and more than 210,000 of these die [1,2]. About 9% of patients with sepsis progress to severe sepsis, and 3% progress to septic shock [3]. Early and accurate diagnosis and risk assessment are pivotal to optimal care of critically ill patients. In an attempt to improve on current sepsis definitions, the PIRO (predisposition, infection, response, organ dysfunction) concept advocates the use of readily measurable circulating biomarkers as an additional tool in the timely assessment and severity classification of septic patients, and in the prediction of mortality [4].

Adrenomedullin (ADM), a peptide with 52 amino acids, has immune modulating, metabolic and vascular actions [5]. It is a potent vasodilator, and its widespread production in tissues helps to maintain blood supply to individual organs [6-8]]. Interestingly, ADM has also bactericidal activity, which is further enhanced by its regulation and modulation of complement activity [9-11]]. Not surprisingly, serum levels of ADM were shown to be increased in sepsis [12]. Quantification of ADM could be helpful in diagnosis and monitoring of sepsis and in prognostication. Unfortunately, the measurement of ADM is technically challenging and reliable measurement is almost impossible because it is rapidly cleared from the circulation [7,8,13,14]. In addition, circulating ADM is masked by a binding protein (complement factor H), making it inaccessible for immunometric analysis [9]. Recently, the more stable mid-regional fragment of pro-adrenomedullin (MR-proADM), comprising amino acids 45–92, which directly reflects levels of the rapidly degraded active peptide ADM, was identified in plasma of patients with septic shock [15].

In the present study our aim was to determine the prognostic value of MR-proADM levels in a previously described, well defined cohort of medical intensive care patients, and to compare it with the prognostic values of previously reported biomarkers (for example IL-6, C-reactive protein [CRP], procalcitonin [PCT]) and of two severity of illness scores (for example Acute Physiology and Chronic Health Evaluation [APACHE] II and Simplified Acute Physiology Score [SAPS] II).

## Materials and methods

### Patients

The present study evaluated 101 consecutive critically ill patients admitted to the medical intensive care unit (ICU) of the University Hospital of Basel (Basel, Switzerland). The primary objective of the study was to determine the prognostic value of endocrine dysfunctions in critically ill patients (the Prognostic Value of Endocrine Dysfunctions in Critically Ill Patients (PEDCRIP) study). Characteristics of the study population and study design, and the definitions used are reported in detail elsewhere [16-20]] and are summarized below. Over a 9-month period 101 consecutive patients, including neutropenic and immunosuppressed patients, admitted to the medical ICU were enrolled. Patients were followed until hospital discharge or death. For the purpose of this study ICU mortality was considered. Data were collected on admission (for example during the first 24 hours), on day 2, and on the day of discharge from the ICU or on the day of death. In patients who died within 24 hours after admission, only data on admission were collected (*n *= 5). Vital signs, clinical status and severity of disease, and laboratory parameters (including MR-proADM levels) were assessed each day, and commonly used physiological scores (APACHE II and SAPS II scores) were calculated. Pulmonary artery catheter was not routinely inserted. When feasible, consent was obtained before enrolment in conscious patients; otherwise, consent was obtained from the patients' next of kin. The study protocol had prior approval from the hospital's ethical review board.

Patients were classified at the time of blood collection as having SIRS, sepsis, severe sepsis or septic shock, as defined according to well known consensus criteria [21,22]. SIRS was characterized by the presence of at least two of the following four clinical criteria: fever or hypothermia (temperature >38°C or <36°C); tachycardia (>90 beats/minute); tachypnoea (>20 breaths/minute or <32 mmHg or the need for mechanical ventilation support); and an altered white blood cell count (>12,000 cells/μl or <4,000 cells/μl) or the presence of >10% band forms. Sepsis was defined as SIRS with an infection. Infection was diagnosed according to standardized criteria or, in case of uncertainty, by an infectious disease specialist. This was done retrospectively based on review of the complete patient charts, results of microbiological cultures, chest radiographs and, when available, postmortem examination findings. Severe sepsis was defined as the presence of sepsis and at least one of the following manifestations of organ failure: hypoxaemia (arterial oxygen tension <75 mmHg); metabolic acidosis (pH <7.30); oliguria (output <30 ml/hour); lactic acidosis (serum lactate >2 mmol/l); and an acute alteration in mental status without sedation (reduction by ≥ 3 points from baseline value in Glasgow Coma Scale score. Septic shock was defined as the presence of sepsis accompanied by a sustained decrease in systolic blood pressure (<90 mmHg or a drop of 40 mmHg from baseline) despite fluid resuscitation and the need for vasoactive amines to maintain adequate blood pressure.

A patient could be classified as being septic and, after adequate treatment, as having infection without SIRS. Because the clinical spectrum from SIRS to septic shock is a fluid continuum that can progress rapidly, patients were classified at the time of blood collection. An isolated microorganism was considered to be pathogenic if it was identified within a 24-hour period before or after the onset of the systemic response. Colonization with bacteria (for example asymptomatic bacteriuria in a patient with bladder catheter without leucocyturia) or postmortem positive blood cultures were disregarded. Microbiological tests and antibiotic therapy were prescribed by physicians on duty in accordance with usual practice, without interference from the research team.

For comparative purposes, MR-proADM values were also measured in 160 age-matched healthy blood donors.

### Assays

Blood was obtained from an indwelling arterial or venous catheter. Results of routine blood analyses (for example complete blood count, serum chemistry including CRP, blood gas analyses) were recorded. The blood was separated into plasma at the time of blood draw and frozen to -70°C. Measurements were done in a blinded manner as a batch analysis.

MR-proADM was detected in EDTA plasma of all patients using a new sandwich immunoassay (B.R.A.H.M.S. Sevadil^® ^LIA; B.R.A.H.M.S., AG, Hennigsdorf/Berlin, Germany), [23]. Briefly, the assay employs two polyclonal antibodies to MR-proADM (amino acids 45–92) and has an analytical detection limit of 0.08 nmol/l. Intra-assay imprecision was under 10% over the entire measuring range, and the functional assay sensitivity (interassay coefficient of variation [CV] <20%) was 0.12 nmol/l. The assay exhibited linear dilution, and pooling of samples or addition of synthetic analyte had no impact on recovery of the analyte. Stability of the analyte (<20% loss of recovery) in EDTA plasma was demonstrated for at least 3 days at room temperature, 14 days at 4°C and 1 year at -20°C. Stability was not compromised by up to four freeze/thaw cycles.

CRP was determined using a routine enzyme immunoassay (EMIT; Merck Diagnostica, Zurich, Switzerland). Its reference range is under 10 mg/l; its intra-assay CVs are 0.9%, 1.4% and 11.4% and interassay CVs are 2%, 2% and 26.3% at CRP levels of 33.6 mg/l, 57.6 mg/l and 150 mg/l, respectively. Serum IL-6 concentrations were measured using a commercially available quantitative sandwich enzyme immunoassay (Pelikine Compact™; CLB, Amsterdam, The Netherlands). Its reference range is under 3.12 pg/ml; its intra-assay CVs are 4.2%, 1.6% and 2.0% and interassay CVs are 6.2%, 3.3% and 3.8% at IL-6 levels of 16.8 pg/ml, 97.7 pg/ml and 186 pg/ml, respectively. PCT levels were assessed using a commercially available immunoluminometric assay (LUMItest PCT; BRAHMS Diagnostica, Berlin, Germany). The lower detection limit of this test is 0.1 μg/l; its intra-assay CVs are 6.3% and 2.7% at PCT levels of 0.4 μg/l and 43.2 μg/l, and its inter-assay CVs are 13.4% and 7.1% at PCT levels of 0.5 μg/l and 34.2 μg/l. The functional detection limit of this assay is 0.3–0.5 μg/l, which is well above the normal reference range in healthy control individuals.

### Statistical analysis

Data are expressed as mean ± standard deviation in the text and as median (range) in figures. Comparison of frequencies was done using the χ^2 ^test. Two-group comparisons were performed nonparametrically using the Mann-Whitney *U *test. For multigroup comparisons Kruskal-Wallis one-way analysis of variance was used, with least square difference *post hoc *evaluation. Receiver operating characteristic (ROC) curves were constructed using MedCalc for Windows (version 7.2.1.0; Broekstraat, Mariakerke, Belgium). Levels that were undetectable were assigned a value equal to the lower limit of detection for the assay. All statistical tests were two tailed, and *P *< 0.05 was considered statistically significant. Correlation analyses were performed using Spearman rank correlation. To estimate the potential clinical benefit of MR-proADM levels, we used likelihood ratio tests to determine whether logistic regression models that included the measurement of MR-proADM and routine clinical parameters (for example APACHE II score) provided a significantly better fit than did logistic regression models limited to APACHE II score alone. This was done using STATA (version 8; STATA Inc., College Station, TX, USA) and Statview (version 4.1; Abacus Concepts, Berkeley, CA, USA) software packages.

## Results

### Patient characteristics

The mean age of the 101 patients (55 men and 46 women) included in this study was 57 ± 15 (range 23–86) years. On admission, the mean APACHE II score was 22 ± 8 points and the mean SAPS II score was 53 ± 18 points. The median length of stay in the ICU was 4 days (range 0.2–60 days) and the mortality rate was 23%. The principal diagnoses of the patients are summarized in Table 1 and the site of infections in Table 2. Sepsis was diagnosed in 58% of the patients (on admission in 53 patients [22 with sepsis, 15 with severe sepsis and 16 with septic shock]; five additional patients developed sepsis during their stay in the ICU). The percentages of patients fulfilling more than two SIRS criteria were as follows: 99% of 101 patients at admission, 96% of 74 patients on day 2, and 68% of 95 patients on the day of discharge or death. Patients who were discharged or died on day 2 were classified into the latter group. The following percentages of patients were classified as having sepsis, severe sepsis, or septic shock: 53% at admission, 60% on day 2, and 36% on the day of discharge or death. The principal site of infection was the lung. In 38 (66%) of the 58 patients with infections, the aetiological microorganism was identified and 14 patients (24%) had bacteraemia. Patients with and patients without infection had similar mortality rates; of the 53 patients admitted with sepsis, severe sepsis, or septic shock, 12 (23%) died of multiple organ failure. Of the 48 patients without infection on admission, 10 (21%) died.

Although optimal fluid resuscitation was done in the initial treatment phase in all patients, 31% of septic patients needed additional treatment with intravenous noradrenaline (norepinephrine). The mean dose of noradrenaline on admission was 8.7 ± 12.1 μg/ml, on day 2 it was 10.1 ± 10.9 μg/ml, and on the day of discharge/death it was 47.2 ± 35.2 μg/ml (*P *< 0.001). Nonsurvivors of severe sepsis and septic shock needed higher doses of noradrenaline than did survivors (5.7 ± 7.8 μg/ml versus 30.5 ± 28.1 μg/ml; *P *< 0.001).

The mean age of the control individuals (82 men and 78 women) was 54 ± 12 (range 23–80) years. Control individuals and patients were well matched with respect to age and sex. In control individuals there was a significant correlation of MR-proADM levels with age (r = 0.53; *P *< 0.001). There was no difference in MR-proADM levels between males and females.

### Mid-regional pro-adrenomedullin and severity of disease

**Figure 1:**
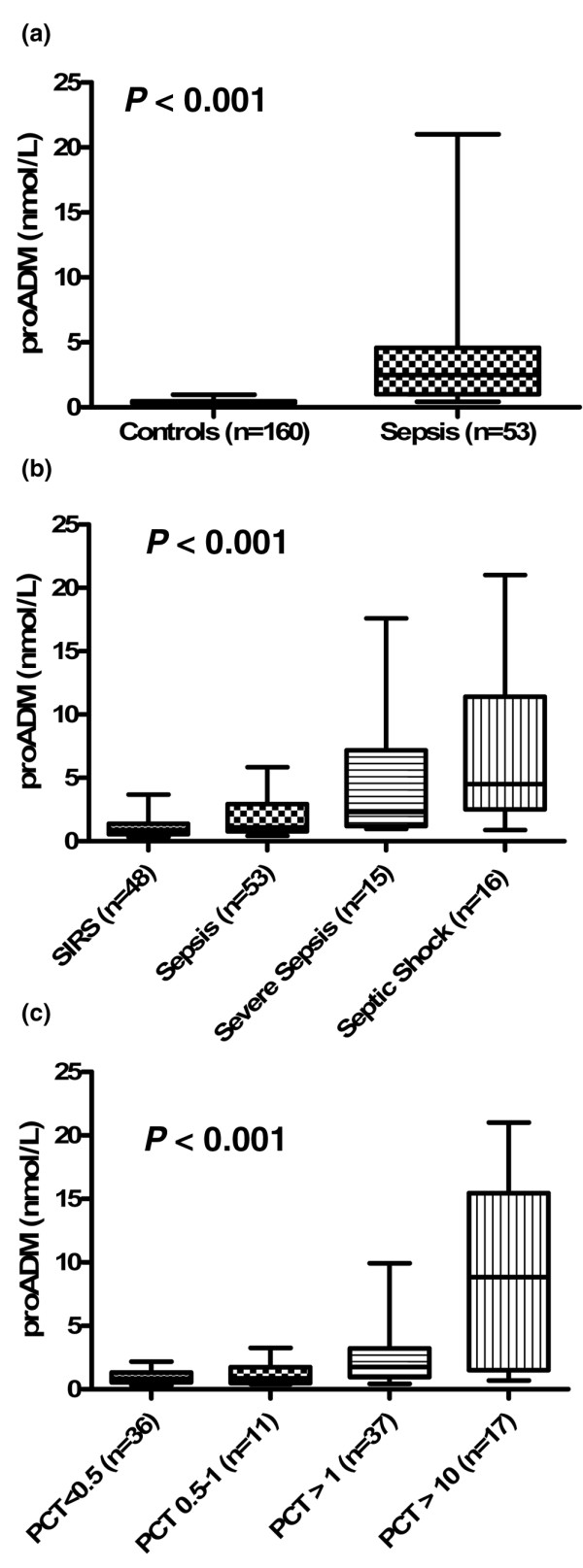
Admission levels of MR-proADM. **(a) **MR-proADM in patients with sepsis versus healthy control individuals. **(b) **MR-proADM in patients without infection (for example SIRS), and in patients with sepsis, severe sepsis and septic shock. **(c) **MR-proADM grouped according to PCT values. Lines denote median values, boxes represent 25–75th percentiles and whiskers indicate the range. The numbers of samples are indicated in parentheses. MR-proADM, mid-regional pro-adrenomedullin; PCT, procalcitonin; SIRS, systemic inflammatory response syndrome.

Figure 1a shows MR-proADM values in healthy blood donors (control individuals) as compared with those in critically ill patients with sepsis (for example sepsis, severe sepsis, and septic shock) on admission. Median (range) values in controls were 0.4 nmol/l (0.21–0.97 nmol/l) as compared with 2.5 nmol/l (0.4–21.0 nmol/l) in patients with sepsis (*P *< 0.001).

In critically ill patients on admission, there was a stepwise increase in MR-proADM levels from patients without infection (for example SIRS) to patients with sepsis, severe sepsis and septic shock (Figure 1b). Median proADM level in patients with SIRS was 1.1 nmol/l (0.3–3.7 nmol/l), in patients with sepsis it was 1.8 nmol/l (0.4–5.8 nmol/l), in patients with severe sepsis it was 2.3 nmol/l (1.0–17.6 nmol/l) and in patients with septic shock it was 4.5 nmol/l (0.9–21 nmol/l). Similarly, circulating MR-proADM levels on admission exhibited a gradual increase with increasing severity of sepsis, as estimated based on PCT levels (Figure 1c). MR-proADM levels on admission exhibited correlations with APACHE II score (r = 0.42; *P *< 0.001), SAPS II score (r = 0.5; *P *< 0.001), PCT (r = 0.65; *P *< 0.001), serum IL-6 (r = 0.53; *P *< 0.001), creatinine (r = 0.32; *P *< 0.001), CRP (r = 0.41; *P *< 0.001) and age (r = 0.3; *P *< 0.01). Correlations with peripheral mean blood pressure and noradrenaline dose on admission were r = -0.28 and 0.3 (both *P *< 0.001). The MR-proADM values in the group of patients receiving noradrenaline were significantly higher than in the group not receiving noradrenaline (5.5 ± 5.6 nmol/l versus 2.1 ± 3.3 nmol/l; *P *< 0.001).

Five patients without infection on admission (for example those with SIRS) developed sepsis during follow up. On admission, median MR-proADM levels in these patients (0.8 nmol/l [0.4–1.5 nmol/l]) were not significantly different from levels in patients who did not develop sepsis (0.9 nmol/l [0.3–3.7 nmol/l]). Of the patients who had sepsis or severe sepsis on admission (*n *= 37), six developed septic shock. Median MR-proADM levels in these patients (4.1 nmol/l [0.8–13.8 nmol/l]) were significantly higher than in patients who stayed stable or did improve (1.5 nmol/l [0.4–17.6 nmol/l]).

### Mid-regional pro-adrenomedullin and outcome of patients with systemic inflammatory response syndrome, sepsis, severe sepsis and septic shock

**Figure 2:**
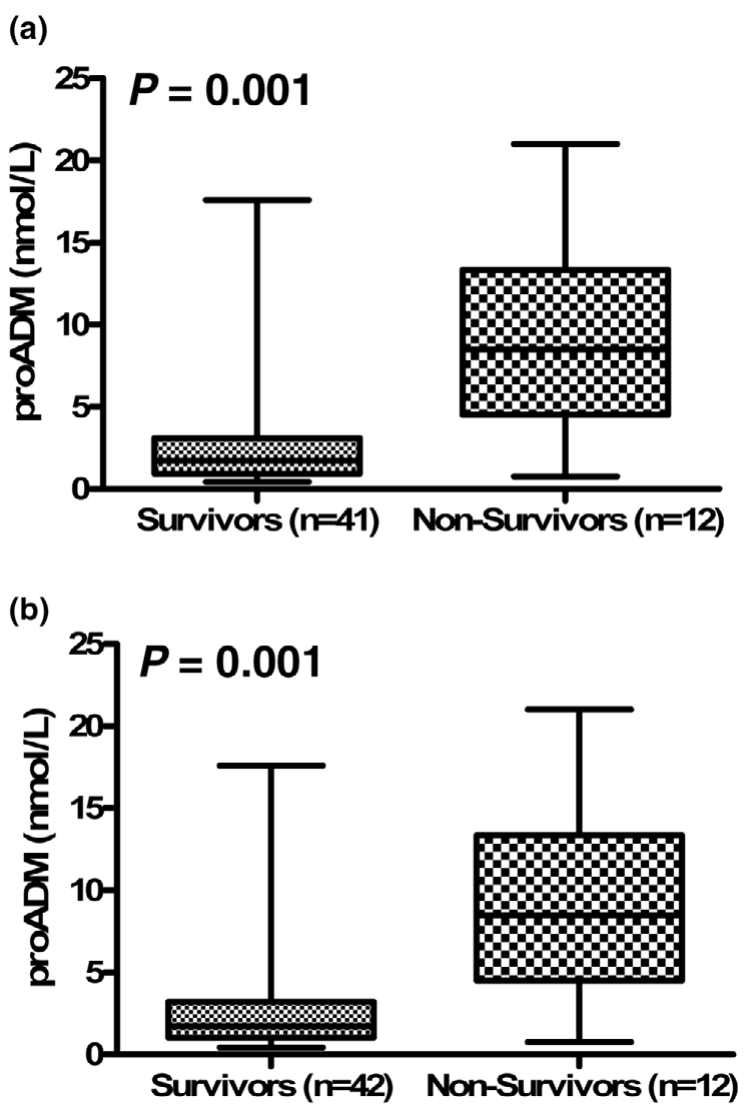
MR-proADM and PCT levels in surviving versus nonsurviving patients. Data from patients on admission are shown. Patients are grouped into **(a) **those with a clinical diagnosis of sepsis based on international guidelines and **(b) **those with circulating PCT levels above 1 ng/ml. Lines denote median values, boxes represent 25–75th percentiles and whiskers indicate the range. MR-proADM, mid-regional pro-adrenomedullin; PCT, procalcitonin.

Figure 2 shows MR-proADM values in survivors as compared with those in nonsurvivors with sepsis, severe sepsis, or septic shock, measured on admission. Patients are grouped into those with a clinical diagnosis of sepsis based on international guidelines (Figure 2a) and those with circulating PCT levels above 1 ng/ml (Figure 2b). The median (range) MR-proADM value on admission in nonsurvivors (8.5 nmol/l [0.8–21.0 nmol/l]) was significantly higher than in survivors (1.7 nmol/l [0.4–17.6 nmol/l]; *P *< 0.001) according to both, sepsis guidelines and ProCT levels >1, respectively. This difference between survivors and nonsurvivors on admission was also significant for IL-6 but not for PCT or CRP (data not shown). In patients without infections, MR-proADM values on admission were not higher in nonsurvivors than in survivors.

**Figure 3:**
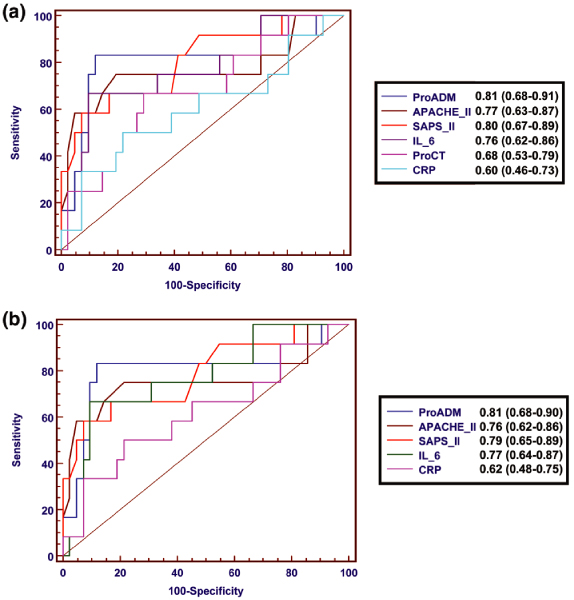
Sensitivity and specificity of various biomarkers and scoring systems in predicting ICU mortality. Shown are ROC plot analyses of the abilities of various biomarkers to predict outcome (for example ICU mortality) in sepsis. Patients are grouped into **(a) **those with a clinical diagnosis of sepsis based on international guidelines and **(b) **those with circulating PCT levels above 1 ng/ml. Sensitivity was calculated with patients who died during their ICU stay and specificity was calculated with ICU survivors. The areas under the ROC curve are given with 95% confidence intervals. ICU, intensive care unit; PCT, procalcitonin; ROC, receiver operating characteristic.

To define the optimal prognostic accuracy for MR-proADM values in septic patients, we performed ROC analysis including only data from patients with sepsis, severe sepsis, or septic shock obtained at admission to the ICU. Sensitivity was calculated in those patients who died during their stay on the ICU, and specificity was assessed in ICU survivors. For comparison, the same ROC analysis was performed with CRP, PCT, IL-6, SAPS II score and APACHE II score. The AUC for MR-proADM on admission was 0.81. Comparisons of the ROC curve for MR-proADM with the ROC curves for the other parameters (for example PCT [*P *= 0.086], CRP [*P *= 0.05], APACHE II score [*P *= 0.64], SAPS II score [*P *= 0.92] and IL-6 [*P *= 0.52]) are shown in Figure 3. Again, patients were grouped into those who had a clinical diagnosis of sepsis according to international guidelines (Figure 3a) and those with circulating PCT levels above 1 ng/ml (Figure 3b), yielding similar results.

The optimal prognostic accuracy for MR-proADM was 3.9 nmol/l. At this cutoff, which is about 10 times the median in the normal population, the sensitivity was 83.3% and the specificity was 87.8%. In comparison, the APACHE II score was also predictive of prognosis but had lower sensitivity and specificity than did MR-proADM. At an APACHE II threshold of 27, the sensitivity was 75% and the specificity was 80.5%. At a cutoff of 25 (which was recommended by the US Food and Drug Administration for the use of drotrecogin alpha), sensitivity was 75.0% and specificity was 70.7%. For the SAPS II score, the optimal prognostic accuracy of 70 points yielded a sensitivity of 58.3% and a specificity of 92.7%.

In addition, likelihood ratio tests were used to compare the fit of predictive models that were based on a routine clinical model (for example APACHE II score) in combination with MR-proADM levels versus the fit of a clinical model based only on APACHE II score. In this model, MR-proADM tended to improve the power of the APACHE II score from an AUC of 0.77 to an AUC of 0.81 (95% confidence interval 0.63–0.99; *P *= 0.50).

## Discussion

We found a significant increase in MR-proADM in the plasma of septic patients as compared with those in healthy controls and in critically ill patients without infections. As a prognostic marker, MR-proADM plasma levels were significantly higher in those patients with sepsis who did not survive than in survivors. The prognostic accuracy tended to be superior to those of other biomarkers such as CRP and PCT, and was in the range of accuracy of IL-6 and commonly used physiological scores as well as of the previously reported pro-atrial natriuretic peptide.

There are two primary mechanisms that might be responsible for the marked increase in circulating MR-proADM and mature ADM in sepsis. First, as a member of the CALC gene family, ADM is widely expressed and extensively synthesized during sepsis, similar to other calcitonin peptides including PCT and calcitonin-gene related peptides [24]. The term 'hormokine' was proposed to encompass the cytokine-like behaviour of hormones, specifically calcitonin peptides, in inflammation and infections [25]. Bacterial endotoxins and proinflammatory cytokines upregulate ADM gene expression in many tissues, both *in vitro *and *in vivo *in rodents and humans [5,26]. In a second potential mechanism, decreased clearance by the kidneys may be responsible in part for increased levels in sepsis [12]. This hypothesis is also supported by a significant correlation between MR-proADM and creatinine levels in the patients included in the present study (r = 0.76; *P *< 0.001). Patients with end-stage renal failure had plasma ADM levels about fivefold greater than those of normal individuals; however, these did not change following haemodialysis [27]. An alternative site of clearance may be the lung. It has been reported that ADM concentrations in the aorta are slightly lower than those in the pulmonary artery during selective catheter sampling [28]. Therefore, impaired removal of circulating ADM during pulmonary circulation resulting from sepsis-associated lung injury may partly contribute to the elevation in plasma ADM levels. However, in the present study MR-proADM levels were similar, irrespective of whether an arterial or a venous catheter was used.

It has been suggested that ADM contributes to the extreme vasodilatation and hypotension associated with septic shock [29]. In the present study, MR-proADM levels exhibited only a weak albeit statistically significant correlation with peripheral blood pressure.

Although it is known that ADM is elevated in sepsis, reported concentrations are much lower than the MR-proADM levels observed in our study [30]. Conceptually, circulating levels of a potent mediator such as ADM are kept within a very narrow range in order to prevent the harmful effects of excessive levels. Hence, in sepsis circulating levels of ADM are only modestly elevated and are not significantly different between patients with SIRS and those with sepsis, prohibiting its use as a diagnostic tool. In contrast, circulating levels of less active precursor peptides are less tightly controlled, and therefore they may vary to a much greater extent between health and disease. Our finding of an ADM precursor facilitates assessment of the actual release of ADM gene products under pathological conditions that involve dysfunction of the cardiovascular system, which in turn may help to improve diagnosis, monitoring and prognostication in these disease states.

Sepsis is a complex syndrome, and the immunological and biochemical situation is heterogeneous among individual patients [31,32]. The APACHE II score was not originally proposed for use in individual outcome prediction in sepsis [33]. Despite their inherent limitations, outcome predictors are nevertheless helpful in identifying those septic patients who are at high risk for death, who are more likely to benefit from intervention [34]. In our opinion, when one is presented with the difficult task of prognostic assessment and making treatment decisions, it is advisable to rely on several parameters that reflect different physiological aspects. Within this context, we proposed the use of biomarkers (for example pro-atrial natriuretic peptide) to aid in the prediction of outcome from sepsis [20]. However, in this task the available biomarkers are far from perfect. For example, IL-6 levels in our study had prognostic value similar to that of MR-proADM levels, but IL-6 levels usually exhibit a sudden and transient rather than sustained increase in sepsis, making measurement of this cytokine and its use as a prognostic marker difficult [35]. Once it is available for use in the routine setting, a proADM assay might prove to be an additional and helpful tool, permitting broader prognostic classification of septic patients.

As is generally recommended, we diagnosed sepsis, severe sepsis and septic shock based on well defined and widely accepted consensus guidelines [21,22]. However, because 'gold standards' for the diagnosis of infection do not exist, ambiguity in clinical classification of critically ill patients remains, both in trials and in routine bedside practice [36,37]. The PIRO concept attempts to improve on current sepsis definitions and considers inclusion of readily measurable circulating biomarkers [4]. An ideal sepsis marker should permit early diagnosis, should inform about the course of disease, and should help one to differentiate bacterial from noninfectious and viral causes of systemic inflammation. It was shown that PCT has some of these features and is helpful in the diagnosis of a septic condition [24,38,39]. Therefore, we also classified MR-proADM levels according to circulating PCT levels, which are independent from the ambiguity associated with clinical sepsis definitions. Importantly, the prognostic value of MR-proADM was similarly independent of the classification system used, increasing the external validity and generalizability of the findings of our study. Thus, based on our data, MR-proADM might be of prognostic value in septic patients, if our findings are confirmed in larger studies.

Our study has limitations. The generalizability of our findings is limited by the fact that only 53 septic patients on admission were included. We consider our results to be preliminary and hypothesis generating. Future studies including more patients and daily follow-up measurements are warranted to validate our results. If our findings can be confirmed, then MR-proADM might become useful as an additional prognostic marker for individual risk assessment of sepsis, and it may become a helpful tool for patient stratification in future intervention trials.

## Conclusion

We propose MR-proADM as a new prognostic biomarker in critically ill patients with different severities of sepsis. Our preliminary data suggest that the ability of MR-proADM to predict outcome is similar to those of the APACHE II and the SAPS II scores.

## Key messages

• 	In septic patients, MR-proADM levels on admission to a medical ICU were able to predict outcome with accuracy similar to that of APACHE II and SAPS II scores.

• 	Because only 53 patients with sepsis on admission were included, the generalizability of our findings is limited, and so our results must be interpreted as preliminary and hypothesis-generating; further studies are warranted to validate our results.

## Abbreviations

ADM = adrenomedullin; APACHE = Acute Physiology and Chronic Health Evaluation; AUC = area under the curve; CRP = C-reactive protein; CV = coefficient of variation; ICU = intensive care unit; IL = interleukin; MR-proADM = mid-regional pro-adrenomedullin; PCT = procalcitonin; ROC = receiver operating characteristic; SAPS = Simplified Acute Physiology Score; SIRS = systemic inflammatory response syndrome.

## Competing interests

NGM, JS and AB are employees of B.R.A.H.M.S., the manufacturer of the proADM assay (Brahms Sevadil^® ^LIA; B.R.A.H.M.S. AG, Hennigsdorf/Berlin, Germany).

## Authors' contributions

BM conceived the study, collected the data, drafted the protocol and supervised the writing of the manuscript. MCC and NGM conducted the analyses and wrote the report. NGM, JS and AB were involved in assay development. SH conducted statistical analyses. All authors read and approved the final manuscript.

## Figures and Tables

**Table 1:** Clinical diagnoses of patients

Diagnosis	Number of patients^a^
Respiratory	61
Pneumonia	33
Chronic obstructive pulmonary disease	14
Acute asthma	3
Bronchial carcinoma	3
Pneumothorax	3
Pharyngeal obstruction	2
Toxic pulmonary oedema	2
Wegener's granulomatosis	1
Cardiovascular	26
Myocardial infarction	12
Heart failure	11
Pulmonary embolism	2
Haemorrhagic shock	1
Abdominal	24
Gastrointestinal bleeding	7
Abdominal infection	6
Urinary tract infection	5
Acute renal failure	3
Hepatic coma	3
Cerebral	21
Ischaemic stroke	5
Subarachnoid haemorrhage	4
Intracerebral haemorrhage	3
Seizures	3
Suicidal intoxication with sedatives	5
Cavernous sinus thrombosis	1
Others	19
Leukaemia	7
Postoperative	6
Diabetic coma	3
Other infections	3

^a^One patient can have more than one diagnosis, and so the total exceeds the absolute number of patients (*n *= 101).

**Table 2:** Site of infection and microbiology

Site of infection/microbiology	Number of patients^a^
Lung	44
*Streptococcus pneumoniae*	6
*Pseudomonas aeruginosa*	5
*Haemophilus influenzae*	3
*Streptococcus pyogenes*	3
*Staphylococcus aureus*	3
*Klebsiella pneumoniae*	2
*Escherichia coli*	2
*Enterobacter *spp.	2
*Streptococcus salivarius*	1
*Legionella pneumophilia*	1
Unknown	16
Urinary tract	6
*Escherichia coli*	5
*Pseudomonas aeruginosa*	1
Abdominal (gastrointestinal tract, liver, bile duct and pancreas)	5
*Clostridium difficile *associated colitis	1
Unknown	4
Others	3
Meningococcal meningitis	1
Sepsis caused by *Torulopsis glabrata*	1
Malaria due to *Plasmodium falciparum*	1

^a^An infection was diagnosed in 58% of the patients (on admission in 53 patients; five additional patients developed sepsis during their stay in the medical intensive care unit).
